# Mechanisms of Alcohol-Induced Damage to the Developing Nervous System

**Published:** 2001

**Authors:** Charles R. Goodlett, Kristin H. Horn

**Affiliations:** Charles R. Goodlett, Ph.D., is a professor in the Department of Psychology, Indiana University-Purdue University, Indianapolis, Indiana, and Kristin H. Horn is a Ph.D. candidate in the Program in Medical Neurobiology, Indiana University School of Medicine, Indianapolis, Indiana

**Keywords:** prenatal alcohol exposure, central nervous system, oxidative stress, mitochondria, growth-arresting factors, embryologic development, alcohol-related intrauterine disorder, alcohol-related neurodevelopmental disorder, necrosis, neurotransmitters, glucose intolerance, cell adhesion molecules

## Abstract

Numerous mechanisms likely contribute to the damaging effects of prenatal alcohol exposure on the developing fetus and particularly the developing central nervous system (CNS). The coexistence of a multitude of mechanisms that may act simultaneously or consecutively and differ among various cell types poses particular challenges to researchers. To study alcohol’s effects on the fetus more easily, investigators have used animal models and tissue-culture experiments. Such approaches have identified numerous potential mechanisms through which alcohol acts on the fetus, many of which result in cell death by necrosis or apoptosis. Among these mechanisms are increased oxidative stress, damage to the mitochondria, interference with the activity of growth factors, effects on glia cells, impaired development and function of chemical messenger systems involved in neuronal communication, changes in the transport and uptake of the sugar glucose, effects on cell adhesion, and changes in the regulation of gene activity during development.

Maternal drinking during pregnancy can adversely affect the outcome of the offspring, with effects ranging from mild cognitive impairment, characterized by impaired mental activities, to full-blown fetal alcohol syndrome (FAS), characterized by growth deficiency, central nervous system (CNS) disorders, and a pattern of distinct facial features. Alcohol can exert these effects both directly, by acting on fetal tissue, and indirectly, by interfering with the maternal support of the growing fetus. Such indirect mechanisms include altering the placenta’s ability to provide the necessary nutrients to the developing fetus. Alcohol also may indirectly harm the fetus by impairing the mother’s physiology. For example, alcoholism may lead to malnutrition or be combined with other drug use.

This article concentrates on the mechanisms underlying alcohol’s direct effects on the fetus. Numerous mechanisms have been suggested as contributing to alcohol-induced fetal damage, particularly deficits in brain function, although none of these mechanisms has been established with certainty. Furthermore, although alcohol itself generally is considered the primary birth-defect-inducing substance (i.e., teratogen), products resulting from alcohol’s breakdown (i.e., metabolism) also may play a role. For example, acetaldehyde—a toxic chemical formed by the breakdown of alcohol in the liver and other tissues—can accumulate in the fetal brain after prenatal alcohol exposure (Hamby-Mason et al. 1997) and may contribute to the development of FAS. Recent literature reviews confirm that no single putative mechanism can account for all the components and variations of the anatomical and behavioral characteristics (i.e., phenotypes) found in children prenatally exposed to alcohol (Abel and Hannigan 1995; Guerri 1998; Maier et al. 1996; Michaelis 1990; Michaelis and Michaelis 1994; Phillips et al. 1989; Schenker et al. 1990; West et al. 1994).

This article reviews some general challenges researchers face when trying to elucidate multiple disease mechanisms and explains the role that animal and tissue culture (i.e., in vitro) studies can play in this research. The article then explores some of the mechanisms that have been implicated in the development of alcohol-induced CNS deficits, which represent the most serious consequences of prenatal alcohol exposure.

## Challenges Associated with the Presence of Multiple Mechanisms

Identifying the mechanisms contributing to alcohol-induced fetal damage is complicated by numerous factors. For example, scientists have not determined the exact cellular and molecular processes involved in normal CNS development, making it difficult to tease apart the effect that alcohol has on this system. In addition, alcohol is known to interact with tissues in a multitude of ways, and those interactions may have both short-term and long-term effects. Finally, each person exhibits a different combination of alcohol-related effects, which is determined by the timing, level, pattern, and duration of the mother’s drinking as well as by genetic factors. This variability makes it difficult to compare alcohol’s effects from one person to the next.

Alcohol’s effects on the developing brain are particularly complex. For certain groups of brain cells, alcohol can lead to cell death, whereas for other cell groups it interferes with cellular functions. Alcohol may even deplete cells through different mechanisms in a given cell population, depending on the developmental stage of the cells (Guerri 1998; West et al. 1994). For example, nerve cells (i.e., neurons) in the fetal brain multiply through a process of cell division and then migrate during development to an appropriate location where they mature to their full form and function. If some groups of those cells are exposed to alcohol during cell division, the generation of new cells may be reduced by an altering of the cell division rate. If exposure occurs at a later stage of development, however, when the cells are no longer dividing, this same population of neurons can be depleted as a result of alcohol-induced cell death.

As brain cells develop, they may change with respect to their susceptibility to alcohol’s effects. This changing susceptibility to alcohol can be illustrated using a neuronal cell type called Purkinje cells that are located in the cerebellum, a brain region involved in motor coordination and motor learning. During cell division, alcohol exposure appears to have minimal effects on the generation of new Purkinje cells. However, at a later stage of development when the cells begin to develop all the features of a mature neuron, Purkinje cells (and perhaps other neurons) are especially vulnerable to cell death.

Neurons may also die because alcohol exposure during one stage of development (e.g., before neurons migrate to their final location) interferes with subsequent developmental stages (e.g., migration or differentiation). For example, cells from an embryonic structure called the cranial neural crest migrate to appropriate locations to form facial cartilage and bone as well as the peripheral nerves that innervate the head and face. (For more information on the embryonic development of the nervous system, including neural crest cells, see the sidebar, p. 179.) In chick embryos, alcohol exposure before the cranial neural crest cells begin to migrate results in excessive cell death of these cells during the period of migration, leading to abnormal facial features modeling effects in humans with FAS (Cartwright et al. 1998).

**Embryonic Development of the Nervous System**

The embryonic development of humans, including the development of the nervous system, is basically the same as in other vertebrates. During early development—approximately 2 weeks after fertilization in humans—a process called gastrulation occurs during which the embryo forms three distinct cell layers that subsequently develop into different body structures:

- The *ectoderm*, which produces the skin and nervous system
- The *endoderm*, which forms the lining of the digestive tract, the respiratory tubes, and associated organs
- The *mesoderm*, which generates the cardiovascular system, bones, muscles, and connective tissue.

Approximately 3 weeks after fertilization in humans, certain cells in the mesoderm (i.e., the notochord cells, which eventually form the spinal column) induce the ectoderm above them to fold upward and form two ridges along the embryo’s midline (see figure). The tops of these ridges, called the neural folds, then curve toward each other until they meet and fuse to form a tube. This is the neural tube that will eventually form the brain and spinal cord (i.e., the central nervous system). The cells originating at the junction of the two neural folds are called the neural crest cells. They will eventually detach and migrate to various locations throughout the embryo to form many vital body structures.

Researchers investigating the causes of fetal alcohol syndrome (FAS) have studied extensively the neural crest cells, because they are particularly sensitive to alcohol-induced injury and cell death. This research has focused particularly on a subset of cells called cranial neural crest cells, which develop, among other structures, into the facial cartilage and bones. Alcohol’s deleterious effects on these cranial neural crest cells during a narrow period of embryonic development most likely cause the characteristic facial features observed in people with FAS.

—Charles R. Goodlett and Kristin H. Horn

**Figure:**
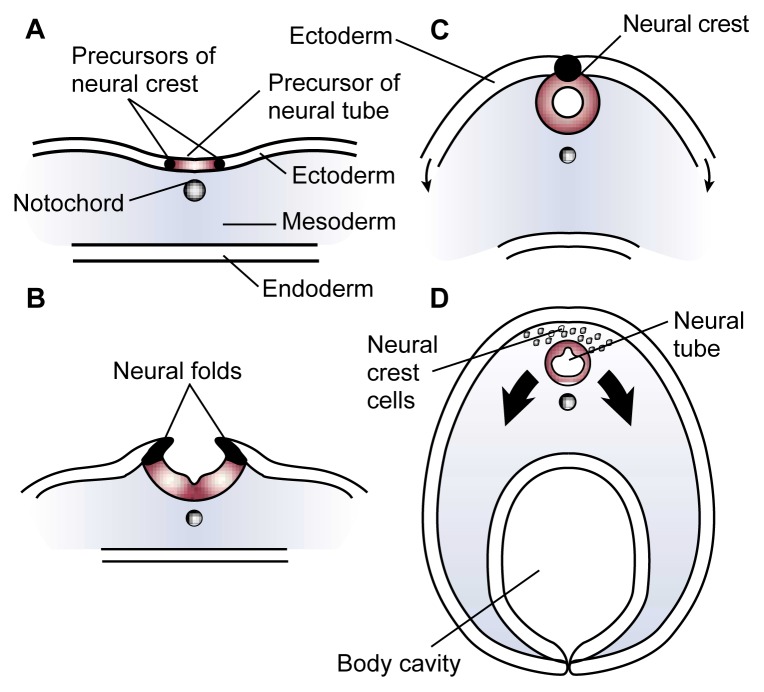
Formation of the neural tube (cross view). Early in an embryo’s development, a strip of specialized cells called the notochord (A) induces the cells of the ectoderm directly above it to become the primitive nervous system (i.e., neuroepithelium). The neuroepithelium then wrinkles and folds over (B). As the tips of the folds fuse together, a hollow tube (i.e., the neural tube) forms (C)—the precursor of the brain and spinal cord. Meanwhile, the ectoderm and endoderm continue to curve around and fuse beneath the embryo to create the body cavity, completing the transformation of the embryo from a flattened disk to a three-dimensional body. Cells originating from the fused tips of the neuroectoderm (i.e., neural crest cells) migrate to various locations throughout the embryo, where they will initiate the development of diverse body structures (D).

These examples demonstrate that at least some consequences of alcohol exposure at different stages of CNS development are caused by different mechanisms (e.g., effects on cell division, on the survival of cells that are migrating after cell division has ended, and on the establishment of mature cell structures and functions). Even at a given developmental stage, different mechanisms of cell death may operate in response to different levels of maternal alcohol consumption and, consequently, fetal blood alcohol concentrations (BACs). Considering these factors, multiple mechanisms leading to alcohol-related fetal damage may operate both simultaneously and sequentially over time.

The presence of such a broad spectrum of potential mechanisms associated with prenatal alcohol exposure makes it difficult to identify alcohol’s effects on specific cells, as well as the consequences of these effects. To facilitate these analyses and to be able to specifically assess alcohol’s effects on specific brain cells, researchers frequently have turned to animal models of FAS or to the study of cells grown in culture (i.e., in vitro models). The benefits and disadvantages of such model systems are discussed in the following section.

## The Role of Animal and in Vitro Models

Researchers have recreated each of the major characteristics of human FAS—facial abnormalities, CNS abnormalities, and growth deficiency—in one or more animal models of developmental alcohol exposure (e.g., chicks, mice, rats, or primates). Such animal models allow investigators to study alcohol’s effects on fetal development in the context of an intact organism while enabling them to control several key factors that influence the type and extent of alcohol-induced structural, functional, and behavioral abnormalities in CNS development (Abel and Hannigan 1995). These factors include the pattern of alcohol exposure (e.g., constant exposure versus bingelike episodes), the BACs produced, the developmental timing and duration of exposure, the cell types or brain regions studied, and maternal and fetal genetic factors (Maier et al. 1996; West et al. 1994).

Because animal studies can be used to mimic the conditions of maternal alcohol consumption during pregnancy and because laboratory mammals have physiological, biochemical, and genetic features in common with humans, the findings from such studies can often provide key information for inferences about effects in humans. Nevertheless, the results should be interpreted with some caution because species differences in vulnerability to alcohol can exist. Furthermore, animal models still do not allow a detailed analysis of alcohol’s actions on individual cells. Such detailed experimental analysis of alcohol’s molecular mechanisms of action is possible, however, using in vitro approaches, in which individual cell types or tissues are grown in tissue culture. These models allow researchers to control and manipulate both the cell and its environment, features that are essential to understanding the mechanisms underlying alcohol’s developmental effects. Even in relatively simple cell culture models, however, multiple harmful effects can occur via a variety of pathways.

The main advantage of in vitro models is that experimental techniques are now available to visualize molecular events during the initial alcohol-tissue interactions and the dynamic course of the pathogenic process in living cells. As a result, in vitro studies are a necessary and powerful tool for discovering alcohol’s molecular actions. At the same time, however, it can be difficult to extrapolate the findings from the molecular actions observed in vitro to the complex mechanisms that simultaneously occur and interact in developing mammalian brains.

The gap between the molecular in vitro data and the analyses of animal models may at least in part be bridged by new techniques for conducting functional molecular analyses of alcohol-related mechanisms in whole organisms. These new techniques include embryo culture methods, as well as manipulation of the animal’s genome (i.e., the total genetic material), such as the generation of mice that lack specific genes—null mutant mice—or that carry foreign genes—transgenic mice. Other emerging technologies, such as genomic and proteomic technology that can be applied both in vivo and in vitro (see textbox), will likely enhance the understanding of mechanisms that damage the developing nervous system. Together, these techniques, which have emerged in tandem with the recent molecular revolution in neuroscience, should yield new insights into the mechanisms of alcohol-induced damage to the developing brain.

**Genomic and Proteomic Techniques Used To Study Alcohol’s Harmful Effects on Development**

Some of alcohol’s harmful effects on the developing organism probably result from alcohol-induced changes in gene expression—the processes through which the genetic information encoded in the genes is converted into gene products (i.e., proteins). Because a large number of genes are active at any given time in a cell, analyses of alcohol-induced changes were previously considered both difficult and time consuming. However, new genomic technologies, such as gene microchip arrays, provide a means to screen the expression of large numbers of genes and identify alcohol-induced changes. This approach allows researchers to evaluate simultaneously changes in the expression of thousands of genes, thereby providing snapshots of alcohol-induced increases or decreases in gene expression. This technique may reveal otherwise undetected patterns of changes across many groups of related genes (i.e., gene families).

Proteomic technology allows the simultaneous separation and identification of a large number of proteins from a target tissue. This approach complements genomic analyses by allowing researchers to detect and identify changes in the production of numerous proteins in specific brain regions following exposure to alcohol.

## Candidate Mechanisms

The experimental approaches described in the previous section—animal models, in vitro models, and new technologies—have helped researchers identify numerous mechanisms that may contribute to alcohol’s detrimental effects on fetal development, as discussed below.

### Cell Death Modes: Necrosis and Apoptosis

**Figure 1:**
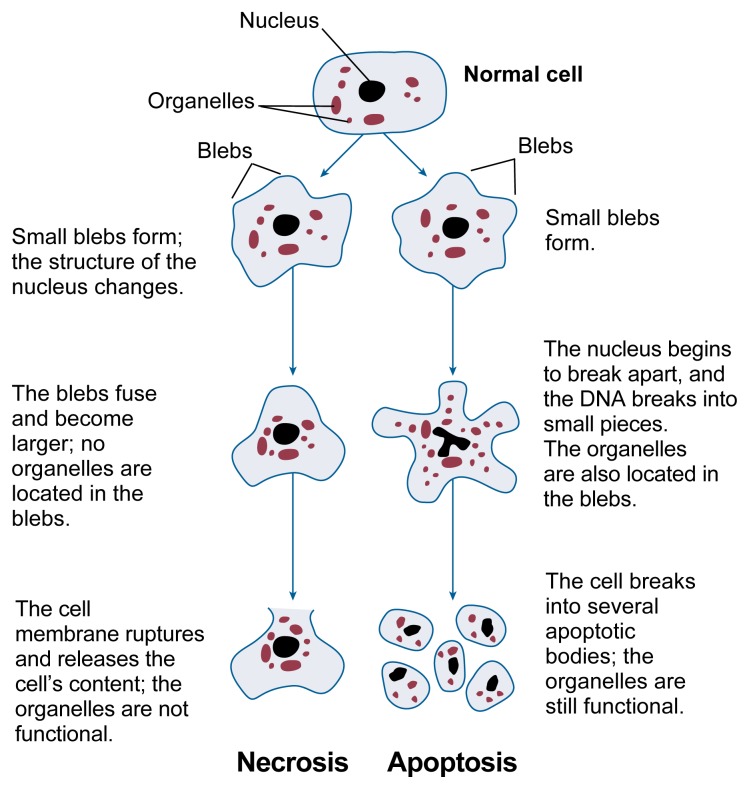
Structural changes of cells undergoing necrosis or apoptosis.

Many actions of alcohol on the developing organism, including the brain, result in cell death. Two general processes of cell death exist, called necrosis and apoptosis. These two processes can be distinguished through different patterns of morphological and biochemical changes during cell death (see figure 1).

Necrosis occurs when neurons are damaged by a trauma or metabolic injury and typically involves the concurrent death of groups of adjacent cells. Cells undergoing necrosis initially swell and their internal components, or organelles, break down. The cells eventually rupture and spill debris that leads to local inflammation. This inflammation can then result in the death of adjacent cells.

Conversely, apoptosis is a form of “cell suicide” that affects only individual cells, leaving adjacent cells intact.^1^ During apoptosis, the cell body shrinks, and the DNA in the nucleus becomes condensed before breaking apart into small fragments. The cell’s organelles remain intact, however. Eventually, the cell breaks up into several smaller bodies that are still surrounded by a membrane. These “apoptotic bodies” then are engulfed and destroyed by scavenging cells. Although apoptosis can progress rapidly once it has been initiated, its onset may be delayed for a time after a toxic insult. At least in some cases, apoptotic cell death appears to involve the activation of a gene-directed program for cellular self-destruction (Bredensen 1996*a*,*b*).

Numerous factors can induce apoptosis of CNS cells, including insufficient blood supply to the brain; dysfunction of the cell’s energy-generating organelles, called the mitochondria; disruption of the normal calcium levels in the cells; and oxidative stress (described in more detail in the following section). Some of these factors can induce both apoptosis and necrosis. Alcohol can also induce apoptosis. This has been demonstrated both in animal models of early alcohol exposure (e.g., the cranial neural crest in embryos [Cartwright et al. 1998]), and in isolated CNS cells grown in culture, including cells from the hypothalamus (De et al. 1994).

A key event occurring in cells undergoing apoptosis is the activation of certain “death-promoting” enzymes called caspases (Bredensen 1996*a*,*b*). Certain caspases act as “executioners” during apoptosis, cutting apart and functionally destroying important proteins in the cell (Cohen 1997). Although diverse molecular signals can initiate apoptosis, the activation of caspases is a crucial step in the progression to cell death. Accordingly, specific inhibitors of caspases have been shown to prevent apoptotic cell death in several experimental models.

Not all cells exposed to one of the cell death-inducing factors mentioned above actually die. Whether a cell lives or dies is determined by the balance between certain proteins that can activate or block apoptosis. Disruption of this balance in favor of proteins encoded by “cell death genes” might be involved in alcohol-induced apoptosis. One well-studied group of cell-death genes is called the bcl-2 family, a group of genes that encode related proteins. Some of these proteins promote apoptosis, whereas others can prevent apoptosis (Merry and Korsmeyer 1997). An increase of apoptosis-preventing members of the bcl-2 protein family may be able to protect a cell against death under various conditions, including exposure to alcohol (Heaton et al. 1999).

### Free Radicals, Oxidative Stress, and Mitochondrial Dysfunction

A key factor that can induce apoptosis (as well as necrosis) is oxidative stress. This term refers to the consequences of having excess levels of free radicals in the cells. Free radicals are highly reactive molecules that may be formed during various biochemical reactions in the cell. Many of these free radicals contain oxygen and are called reactive oxygen species (ROS). Typically, the levels of ROS and other free radicals are controlled by various scavenger molecules, known as antioxidants, that are normally found within the cell and which eliminate free radicals. If ROS levels exceed the cell’s ability to eliminate them, however, or if the normal antioxidant levels within the cell are reduced due to a toxic insult such as alcohol, then oxidative stress can occur. This oxidative stress can cause damage to cellular components, such as membranes, DNA, and proteins. Moreover, oxidative stress can induce cell death processes through several mechanisms, including the release of apoptosis-inducing factors (Bredensen 1996*a*,*b*).

Alcohol can induce oxidative stress through several mechanisms. For example, certain pathways of alcohol metabolism result in the generation of ROS. Moreover, alcohol may reduce antioxidant levels. Experimental evidence suggests that these factors may contribute to alcohol-induced cell damage and cell death in the fetus (Guerri 1998; Henderson et al. 1995; Kotch et al. 1995). Treatment with antioxidants appears to ameliorate alcohol-induced damage in animal models (Heaton et al. 2000). Therefore, this line of investigation may have important implications for clinical intervention.

The alcohol-induced formation of excess levels of ROS also can damage cells and induce cell death by interfering with the function of the mitochondria—organelles surrounded by a membrane that are found in all cells and which generate most of the cell’s energy. The mitochondria serve an additional crucial function because they store calcium and regulate the calcium levels in the cell, which is particularly critical in neurons. The controlled flow of calcium from the fluid surrounding the neuron into the neuron’s interior is one of the key steps in the process of chemical communication between neurons. To ensure accurate neuronal function, calcium levels inside the neuron must be tightly regulated. Furthermore, excessive internal calcium concentrations can be toxic to neurons (Choi 1995). Therefore, the ability of the mitochondria to actively sequester calcium is vital for maintaining neuronal function and survival.

Oxidative stress, such as the alcohol-induced formation of excess ROS levels, can also be associated with disturbed mitochondrial function, including the mitochondria’s ability to regulate internal calcium levels. Mitochondrial dysfunction can lead to both necrosis and apoptosis (Kroemer et al. 1997). When mitochondria become dysfunctional, they can undergo a process called mitochondrial permeability transition (MPT). During this process, large holes open in the mitochondrial membrane through which the mitochondria release their contents, including calcium and a molecule called cytochrome c, into the fluid that fills the neuron. Both calcium and cytochrome c can activate caspases, which, as mentioned in the previous section, play a role in apoptosis. In addition, the MPT process plays a pivotal role in necrosis, further contributing to the deleterious consequences of alcohol-induced oxidative stress.

### Growth Factors Regulating Cell Proliferation and Survival

Alcohol also can damage the fetal brain by a mechanism in which the generation of new cells in the cerebral cortex is hindered during development. New neurons are formed in two specific areas of the developing brain; from those cell proliferation zones, the new cells migrate to their final locations in the mature brain.

Alcohol can alter the speed at which the cells divide (Miller 1996). Alcohol can also interfere with the activity of growth factors that regulate cell proliferation and survival. Loss of normal growth factor signaling can also interfere with or prevent normal growth and development. Numerous growth factors are needed for cell division to proceed normally, including two factors called insulinlike growth factors (IGF) I and II. Both IGF-I and IGF-II exert their effects by binding to protein molecules called IGF-I receptors on the cell surface. Alcohol can interfere with the activity of the IGF-I receptor. As a result, IGF-I still binds to its receptor, but the receptor’s signaling function is blocked, and IGF-I-mediated cell division cannot proceed (Resnickoff et al. 1993). This example demonstrates that alcohol can prevent the normal production of CNS cells by interfering with the growth factors that regulate cell division.

Alcohol also may induce cell death by inhibiting several growth factors that support cells that have attained their final function (i.e., that are differentiated) and no longer divide. For example, IGF-I and the IGF-I receptor also play a role in the survival of nondividing cells and can prevent apoptosis in several models of cell death. Similar to the situation in dividing cells described above, alcohol can inhibit the IGF-I receptor in nondividing cells, thereby preventing the survival of those cells (Cui et al. 1997; Zhang et al. 1998).

### Effects on Glia Cells

Normal brain development and function require not only neurons, but also non-neuronal cells, called glia, that support the growth and development of the neurons. Various types of glial cells with specialized functions exist. For example, migration of newly formed neurons to their final location in the developing brain requires the presence of cells called radial glia, which serve as elongated cellular “tracks” that direct neurons to their appropriate destinations. Once all neurons have migrated to their final locations, the radial glial cells normally change into another type of glial cell—star-shaped astrocytes.^2^ After prenatal alcohol exposure, however, radial glia may become astrocytes prematurely (Miller and Robertson 1993). As a result, neurons generated toward the end of the neuronal migration period, which normally would migrate to the outer layers of the cerebral cortex, lose their radial glial guides, stop migrating, and end up in abnormal positions. This model could help explain the abnormal positioning of neurons in the cortex observed after developmental alcohol exposure.

Both animal models and in vitro studies found that alcohol exposure alters several aspects of astrocyte structure and function. Thus, alcohol exposure can reduce the overall number of astrocytes in the cortex, reduce or delay the production of the proteins that give the astrocytes their characteristic shape, and interfere with the cells’ production of or response to specific growth factors. Such alcohol-induced changes in astrocyte development and function could have serious consequences on neuronal migration and survival and on the correct formation of connections among neurons.

### Effects on Development of Neurotransmitter Systems

**Figure 2:**
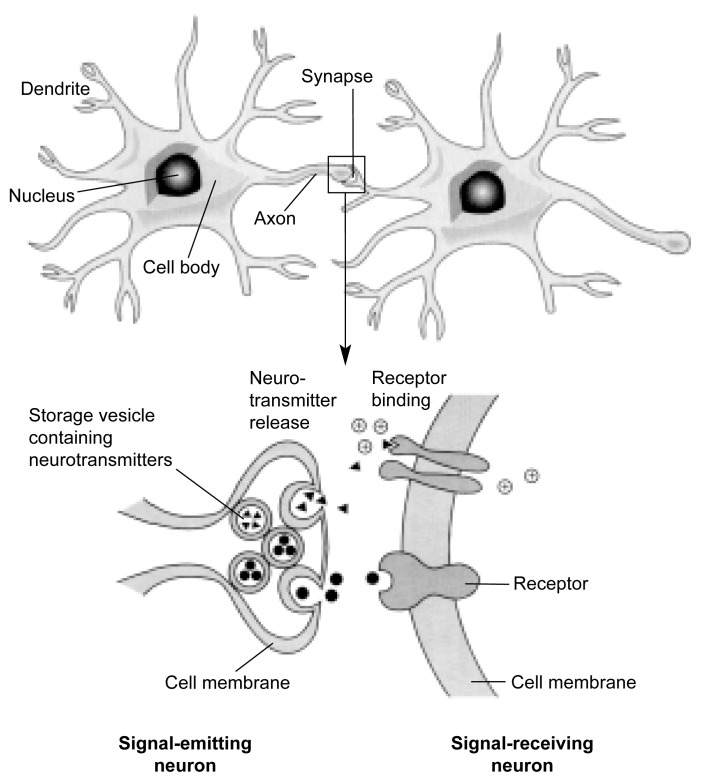
Structural features of a typical nerve cell (i.e., neuron) and synapse. This schematic drawing depicts the major components of a typical neuron, including the cell body with the nucleus; the dendrites that receive signals from other neurons; and the axon, which relays nerve signals to other neurons at a specialized structure called a synapse (see inset). When the nerve signal reaches the synapse, it causes the release of chemical messengers (i.e., neurotransmitters) from storage vesicles. The neurotransmitters travel across a minute gap between the cells and then interact with protein molecules (i.e., receptors) located in the membrane surrounding the signal-receiving neuron. This interaction causes biochemical reactions that result in the generation, or prevention, of a new nerve signal, depending on the type of neuron, neurotransmitter, and/or receptor involved.

Another important mechanism through which alcohol adversely affects the structure and function of the developing brain is by interfering with the activity of neurotransmitters—brain chemicals that allow the transmission of nerve signals from one neuron to the next. This transmission occurs at the junction between two neurons called a synapse. At this juncture, the part of the neuron that conducts nerve signals away from the neuron’s body (i.e., the axon) interacts with the branching extensions (i.e., dendrites) of a neighboring neuron that receives the nerve signal (see figure 2 for the structure of a typical neuron and synapse). During this transmission process, the neurotransmitters are released from storage vesicles at the end of the axon of the signal-emitting neuron and travel across a small gap to the signal-receiving neuron. There, the neurotransmitters interact with specific receptors to induce biochemical reactions in the signal-receiving neuron that promote or prevent the generation of a new nerve signal. Numerous neurotransmitters exist, and some of these (e.g., glutamate, serotonin, and gamma-aminobutyric acid [GABA]) also help organize the CNS during fetal development. Prenatal alcohol exposure can alter the functions of these neurotransmitter systems, particularly the glutamate and serotonin systems.

#### Glutamate

To exert its actions, glutamate interacts with several receptors, including one called the NMDA receptor. During brain development, the interaction of glutamate with the NMDA receptor appears to be critical for stabilizing synapses that have been formed during sensory or other behavioral experiences. Developmental alcohol exposure can reduce the number and/or function of NMDA receptors both during early development and during subsequent developmental stages (Costa et al. 2000; Rema and Ebner 1999). Through this mechanism, alcohol could affect the function of numerous neurotransmitter systems. This could play a major role in the cognitive and behavioral deficits associated with FAS.

#### Serotonin

Another important neurotransmitter that helps regulate brain development early in life is serotonin. The growth of serotonin-releasing (i.e., serotonergic) neurons into the brain area that eventually develops into the cortex appears to be a critical step in cortical development (Whitaker-Azmitia et al. 1996). Developmental alcohol exposure in rats significantly delays the development of the serotonin system and alters the normal interaction between serotonin and its target sites during periods that are likely essential for normal brain development. One mechanism through which alcohol may delay the development of the serotonin system is by interfering with the interactions between developing serotonergic neurons and nearby astrocytes that support the growth and development of those neurons (Kim and Druse 1996; Eriksen et al. 2000). The growth-promoting activity of serotonin and alcohol’s effects on this activity needs to be further elucidated. In addition, researchers still need to characterize how the alcohol-induced deficits in early embryonic growth of serotonergic neurons into the developing cortex affect brain organization and the function of target regions in the cortex.

### Excitotoxicity

Neuronal death also can be induced by excess activity of certain neurotransmitters, including glutamate. This phenomenon, which is called excitotoxicity, may also contribute to alcohol-related damage to the developing brain (Michaelis 1990; Michaelis and Michaelis 1994). Under certain conditions, when glutamate interacts with the NMDA receptor, it causes calcium to flow into the signal-receiving neuron. As mentioned earlier in this article, such a calcium influx is a powerful regulator of the activity and function of a neuron. In the fetus, the calcium influx generated at the NMDA receptor is an important signal in neuron development, synapse formation, and mechanisms of learning, all of which are crucial to the brain’s ability to adapt to its environment. Excessive activation of the NMDA glutamate receptor, however, can lead to dangerously high calcium accumulation inside the neuron (Choi 1995). If sufficiently severe or prolonged, the rise in intracellular calcium can lead to cell death by either apoptosis or necrosis (Choi 1995; Kroemer et al. 1997; Pang and Geddes 1997).

Conditions of excitotoxicity can occur during withdrawal from high levels of alcohol and may thereby contribute to alcohol-induced damage to the fetal brain, particularly when the mother binge drinks (Thomas and Riley 1998). In these cases, the fetus experiences periods of heavy alcohol exposure, followed by withdrawal episodes. High levels of alcohol acutely inhibit NMDA receptor function. During withdrawal after a binge-drinking episode, however, glutamate stimulation of NMDA receptor activity increases temporarily and may lead to excitotoxicity (Thomas et al. 1997). Although some experimental support exists for the potential contribution of withdrawal-related events to alcohol-induced fetal brain damage (Thomas et al. 1997), including the potential role of excitotoxicity, this hypothesis requires more research.

### Glucose Transport and Uptake

Some of the harmful effects of prenatal alcohol exposure also may be associated with alcohol-induced disruption of the brain’s utilization of the sugar, glucose. Glucose has several crucial functions in the body, including the brain. First, it serves as an energy source in all cells. Second, it is used in the production of various important types of molecules, including DNA and RNA building blocks (i.e., nucleic acids), fat molecules (i.e., lipids), certain hormones (i.e., steroids), and certain neurotransmitters.

To enter cells from the blood and fulfill its functions, glucose must cross the cell membrane. To this end, most mammalian cells contain specific glucose transporter proteins designated GLUT1 through GLUT7. The principal glucose transporter proteins of the brain are GLUT1 and GLUT3. In cultured rat neurons and astrocytes, short-term alcohol exposure reduced cellular glucose uptake as well as the levels of glucose transporter proteins (Hu et al. 1995). Similarly, prolonged prenatal exposure of rats to alcohol reduced both glucose uptake and GLUT1 gene expression (Singh et al. 1992). Because of the central role that glucose plays in the body, alcohol-induced changes in glucose transport have broad implications and must be considered as an important potential contributor to both growth deficiency and CNS damage associated with prenatal alcohol exposure.

### Effects on Cell Adhesion

Yet another mechanism through which alcohol may interfere with normal brain development is by reducing cell adhesion. Neurons must establish cell-to-cell contact during growth and development in order to survive, migrate to their final destination, and develop appropriate connections with neighboring cells. Numerous cell adhesion molecules (CAMs) assist in various aspects of this process. Defects in one particular CAM called L1 can lead to abnormal brain development in humans, characterized by mental retardation, complete absence of the corpus callosum, and abnormal development of the cerebellum. These brain abnormalities are similar to those found in patients with FAS, suggesting that prenatal alcohol exposure also may affect the L1 molecule and thereby contribute to several aspects of the FAS phenotype. This hypothesis is supported by findings that when cultured brain cells are exposed to low levels of alcohol—less than 0.05 percent—the L1-mediated clumping together of the cells is inhibited (Ramanathan et al. 1996).

In an important recent extension of the analysis of alcohol’s cell adhesion effects, researchers demonstrated that this inhibitory effect was specific to certain types of alcohol molecules. The alcohol in alcoholic beverages is chemically known as ethanol. Researchers found that only certain alcohol molecules, such as ethanol, interfere with L1-mediated cell adhesion. Conversely, other types of alcohol molecules, such as a molecule called octanol, actually block ethanol’s effect on cell adhesion in tissue cultures (Wilkemeyer et al. 2000). Octanol even prevented the harmful effects of ethanol on mouse fetuses grown in culture (Chen et al. 2001), suggesting that ethanol’s effect on cell adhesion is an important contributor to the harmful consequences of prenatal alcohol exposure.

### Altered Developmental Regulation of Gene Expression

Another candidate mechanism through which prenatal alcohol exposure could damage the CNS and lead to such devastating consequences as FAS is through interfering with the normal regulation of the genes that control brain development. Researchers have not yet been able to elucidate these processes, leaving a major gap in their understanding of candidate mechanisms underlying FAS. (One exception is the research on the mechanisms contributing to the characteristic facial features associated with FAS, which is described in the textbox below.) Although investigators have identified genes whose expression is altered by alcohol in vitro, studies of alcohol’s effects on gene expression as it relates to the development of various body structures and the CNS are still in their infancy. Detailed studies of alcohol-induced changes in gene expression during critical periods of development constitute one of the highest priorities for new research. Cutting-edge technologies, such as the gene microchip array and proteomic technologies, may provide the means to make rapid advances on this frontier in the near future.

**Retinoic Acid’s Possible Link to FAS’s Distinct Facial Features**

One of the hallmarks of fetal alcohol syndrome (FAS) is a distinct set of facial abnormalities, including small eye openings, a low nasal bridge, a short nose, a small midface, a thin upper lip, and a flat groove between the nose and upper lip (i.e., a flat philtrum). During embryonic development, the facial cartilage and bone responsible for these craniofacial features (as well as certain central nervous system cells) are derived from a subset of embryonic cells called cranial neural crest cells. These cells appear to be particularly sensitive to alcohol. Alcohol’s teratogenic effects on the development of craniofacial structures may involve a reduced production of the compound retinoic acid during a narrow period of early embryonic development.

Derived from vitamin A, retinoic acid is essential for the normal development of various tissues and organs in vertebrates, including the development of neural crest cells into craniofacial features. Retinoic acid acts through specific receptors, some of which turn on or turn off the genes that regulate the timing, coordination, and sequencing of various steps in the development of craniofacial features as well as of certain brain regions. Severe vitamin A deficiency or insufficient retinoic acid formation can produce birth defects, and alcohol can prevent or reduce the production of retinoic acid in the brain. This disruption in the control and timing of retinoic acid-mediated gene regulation may be a key component in alcohol’s harmful effects on the fetus.

## Summary

Alcohol exposure during development has numerous structural and functional effects on the developing fetus, especially the brain. The pattern and severity of these effects depend on the dose, timing, pattern, and duration of the alcohol exposure. Furthermore, the vulnerability to alcohol-induced damage varies across cell types and tissues as well as across stages of fetal development. To date, no global mechanism of alcohol-induced damage to embryonic or fetal brain development has been established, and it is highly unlikely that a single mechanism can account for the various components of the FAS phenotype. The different candidate mechanisms may also have different thresholds in terms of the BACs required for their activation. To gain a comprehensive understanding of the molecular mechanisms of alcohol-induced damage, researchers must determine how the initiation and progression of each type of damage changes with the BACs achieved and the developmental timing of exposure.

The insights gained to date provide numerous opportunities for researchers from various disciplines to share their expertise using state-of-the-art tools of cellular, molecular, and developmental neurobiology to explore the basic causes and consequences of prenatal alcohol exposure. Several strong candidate mechanisms have been identified that should stimulate vigorous research efforts and that likely will provide insight into the development of FAS. The search for additional mechanisms can be guided and informed both by good animal models and by in vitro studies. The identification of new mechanisms and their respective contributions to alcohol-induced fetal damage should accelerate the development of rational approaches to the diagnosis, treatment, and prevention of alcohol-related birth defects. This knowledge also should provide powerful information for effective public education and counseling of alcohol-dependent women of childbearing age.

## Footnotes

1 Although apoptosis has been implicated in a broad range of CNS disorders, including Alzheimer’s disease and amyotrophic lateral sclerosis (ALS, or Lou Gehrig’s disease), it is important to note that apoptosis is not always bad for the organism. In fact, elaborately regulated apoptotic cell death is also critical for normal CNS formation during development.

2 Astrocytes provide structural support for neurons.

## GLOSSARY

Apoptosis: A type of cell death that occurs in individual, damaged cells and during which cells break down in a form of cell suicide as a result of biological processes within the cell; also called programmed cell death (because it occurs during normal development).
Astrocyte: A type of *glia* cell.
Cerebellum: An area at the base of the brain involved in the maintenance of posture, balance, and coordination.
Corpus callosum: A bundle of nerve fibers connecting the brain’s hemispheres.
Cortex (or cerebral cortex): The outer layer of gray matter covering the surface of the forebrain that contains the brain regions controlling sensory, motor, perceptual, emotional, and higher cognitive processes.
Differentiation: The process through which unspecialized embryonic cells or tissues are modified and altered to achieve specific physical forms, physiological functions, and chemical properties.
Enzyme: A protein that facilitates chemical reactions.
Excitatory neurotoxin: Any substance that damages nerve cells by causing excessive nerve cell activity.
Free radicals: Highly reactive molecules that cannot exist in a free state for a prolonged period and which can cause cell damage; include reactive oxygen species.
Gene expression: The processes through which the genetic information contained within a gene on the DNA is converted into a gene product (e.g., a protein).
Glia cell: A type of cell in the central nervous system that serves diverse support functions for the development and function of the nerve cells.
Glucose: A type of sugar molecule that is the main energy source for most cells and which is needed to form the building blocks of DNA, fat molecules, and certain hormones.
Mitochondria: *Organelles* that generate energy for the cell’s metabolic processes; also help maintain calcium levels in nerve cells.
Necrosis: A type of cell death that occurs in groups of cells in response to disease or injury in which cells swell and rupture, typically resulting in inflammation.
Organelle: A functional component of a cell; each organelle has its own membrane and specialized function.
Oxidative stress: The end result of an imbalance between *free radicals* and substances that scavenge those reactive species (i.e., antioxidants); can lead to cell damage.
Pathogenesis: The source or cause of an illness or abnormal condition.
Phenotype: The observable characteristics (e.g., physical appearance or behavior) of an organism.
Receptor: A protein molecule on the surface of a cell that interacts with a specific chemical messenger, such as a neurotransmitter or hormone.
Retinoic acid: A chemical derived from retinol (i.e., vitamin A) that is necessary for normal embryonic development, including the development of a certain cell type, the cranial neural crest cells, into head and facial structures.
Teratogenic: That which causes abnormalities during development.
